# Renin-Angiotensin System and Sympathetic Neurotransmitter Release in the Central Nervous System of Hypertension

**DOI:** 10.1155/2012/474870

**Published:** 2012-11-21

**Authors:** Kazushi Tsuda

**Affiliations:** ^1^Cardiovascular and Metabolic Research Center, Kansai University of Health Sciences, Osaka 590-0482, Japan; ^2^Division of Cardiology, Department of Medicine, Wakayama Medical University, Wakayama 641-8509, Japan

## Abstract

Many Studies suggest that changes in sympathetic nerve activity in the central nervous system might have a crucial role in blood pressure control. The present paper discusses evidence in support of the concept that the brain renin-angiotensin system (RAS) might be linked to sympathetic nerve activity in hypertension. The amount of neurotransmitter release from sympathetic nerve endings can be regulated by presynaptic receptors located on nerve terminals. It has been proposed that alterations in sympathetic nervous activity in the central nervous system of hypertension might be partially due to abnormalities in presynaptic modulation of neurotransmitter release. Recent evidence indicates that all components of the RAS have been identified in the brain. It has been proposed that the brain RAS may actively participate in the modulation of neurotransmitter release and influence the central sympathetic outflow to the periphery. This paper summarizes the results of studies to evaluate the possible relationship between the brain RAS and sympathetic neurotransmitter release in the central nervous system of hypertension.

## 1. Introduction

There is increasing evidence to suggest that sympathetic nervous activity in both central and peripheral nervous systems may play a major role in the regulation of blood pressure, and that hypertension is accompanied characteristically by increased sympathetic nervous activity in both humans and animal models [1, 2]. Pharmacologic studies have demonstrated that depletion of central and peripheral catecholamine stores could prevent or attenuate the development of hypertension [3, 4]. The concept on the release of sympathetic neurotransmitters from nerve endings has been considerably refined by the demonstration of multiple presynaptic receptors, which were shown to either facilitate or inhibit their release [5, 6]. The renin-angiotensin system (RAS) including angiotensin receptors is widely distributed in the brain [7–9]. It is proposed that the RAS facilitates the sympathetic nervous system and that angiotensin-converting enzyme (ACE) inhibition and angiotensin receptor blockade are antiadrenergic [10, 11]. However, the interaction between the brain RAS and sympathetic nervous system are not fully understood. In the present paper, we discuss the relationship between the brain RAS and sympathetic neurotransmitter release and further evaluate the role of RAS in the regulation of sympathetic nerve activity in the central nervous system of hypertension. 

## 2. Amount of Norepinephrine Release in the Central Nervous System of Hypertension

Augmented norepinephrine (NE) release and catecholamine synthesis as well as tyrosine hydroxylase gene expression have been reported at central sites related to blood pressure regulation in adult spontaneously hypertensive rats (SHR) [12, 13]. In a study presented earlier, Qualy and Westfall [14] observed that stimulation-evoked NE release from paraventricular hypothalamic nucleus in hypertensive rats was significantly increased in comparison with normotensive rats. We have been demonstrating that the stimulation-evoked NE release from hypothalamic slices was significantly greater in SHR than in normotensive Wistar-Kyoto (WKY) rats, suggesting that the release of NE from the hypothalamus may contribute to the elevated sympathetic nerve activity in hypertension [15–22]. It was also demonstrated that, by using electrophysiological method, higher discharge rates were detected in neurons of the rostral ventrolateral medulla (RVLM) of neonatal SHR [23]. On the other hand, the stimulation-evoked NE release in the slices of whole medulla oblongata was not significantly different between SHR and WKY rats [24–29]. Recently, Teschemacher et al. [30] demonstrated that although overall electrophysiological characteristics of C1 and A2 catecholaminergic neurons in the brain were compatible between SHR and WKY rats, the angiotensin II- (Ang II-) induced Ca^2+^-mobilization was reduced in A2 neurons of SHR. Because A2 neurons are a part of an antihypertensive circuit, the reduced sensitivity of the A2 cells to Ang II might further compromise their homeostatic role in SHR. There might be regional differences in the amount of NE release in the central nervous system of hypertensive models. 

In human hypertensive subjects, Esler et al. [31] studied the brain NE release and its relation to peripheral sympathetic nervous activity by using transmitter washout method. They showed that overall NE overflow into the internal jugular vein was significantly increased in subjects with essential hypertension compared with normotensive subjects. The finding indicated that central sympathetic tone might be activated in essential hypertension, although precise mechanisms regulating central sympathetic neurotransmitter release in human hypertension is not fully understood. 

## 3. Role of Renin-Angiotensin System in the Regulation of Norepinephrine Release in the Central Nervous System 

### 3.1. Angiotensin II

It was shown that angiotensin I (Ang I) and Ang II injected to the central nervous system significantly elevated blood pressure [32]. Davern and Head [33] showed that the chronic subcutaneous infusion of Ang II caused rapid and marked neuronal activation in circumventricular organs, such as subfornical organ, the nucleus of the solitary tract, paraventricular nucleus, and supraoptic nucleus. In an in vitro study presented earlier, Garcia-Sevilla et al. [34] demonstrated that Ang II facilitated in a concentration-dependent manner the potassium-evoked NE release in the rabbit hypothalamus, which was antagonized by saralasin. The result indicated that the increase in NE release might be mediated through presynaptic angiotensin facilitatory receptors on noradrenergic nerve terminals. It was also shown that Ang II increased the potassium-evoked NE release from slices of rat parietal cortex, and that the effect was blocked by saralasin, but not by the Ca channel blocker, nimodipine [35]. Moreover, the facilitative action of Ang II on NE release might be pronounced in the hypothalamus of SHR compared with normotensive rats [36]. In a microdialysis study, Qadri et al. [37] showed that intracerebroventricular administration of 100 ng of Ang II increased blood pressure and NE release in anterior hypothalamus of conscious rats, which was antagonized by the Ang II receptor blocker. By using the similar method, Stadler et al. [38] also reported that Ang II led to significant dose-dependent increases of NE release in the paraventricular nucleus. 

Several lines of evidence demonstrate that function and signaling of the angiotensin type 1 (AT_1_) receptors are quite different from the angiotensin type 2 (AT_2_) sites and that these receptors may exert opposite effects on blood pressure regulation [39]. Gelband et al. [40] demonstrated that neuronal AT_1_ receptors might have a pivotal role in NE neuromodulation, and that evoked NE neuromodulation might involve AT_1_ receptor-mediated, losartan-dependent, rapid NE release, inhibition of potassium-channels and stimulation of Ca^2+^-channels. Furthermore, they proposed that AT_1_ receptor-mediated enhanced neuromodulation might involve the Ras-Raf-MAP kinase cascade and lead to an increase in NE transporter, tyrosine hydroxylase, and dopamine *β*-hydroxylase mRNA transcription. On the other hand, neuronal AT_2_ receptors might signal via a Gi-protein and be coupled to activation of PP2A and PLA2, and stimulation of potassium-channels. Nap et al. [41] showed that prejunctional AT_1_ receptors might belong to the AT_1B_ receptor subtype because AT_1B_ receptor inhibition by high concentrations of PD 123319 could suppress the Ang II-augmented noradrenergic transmission. Gironacci et al. [36] demonstrated that Ang-(1-7), which is synthesized by angiotensin converting enzyme 2 (ACE2), significantly decreased the potassium-induced NE release in the hypothalamus of SHR by stimulating the AT_2_ receptors. In addition, they showed that the inhibitory effect of Ang-(1-7) on NE release was blocked by the nitric oxide (NO) synthase inhibitor and the bradykinin (BK) B_2_ receptor antagonist. The finding indicated that Ang-(1-7) reduced NE release from the hypothalamus of SHR via the AT_2_ receptors, acting through a BK/NO-mediated mechanism. Recent findings have also revealed that the Mas oncogene may act as a receptor for Ang-(1-7) [42–44]. It is strongly suggested that activation of the ACE2-Ang-(1-7)-Mas axis might act as a counterregulatory system against the ACE-Ang II-AT_1_ receptor axis [42–44]. 

Bourassa et al. [45] demonstrated that AT_1_ receptor binding in both RVLM and caudal ventrolateral medulla as well as dorsomedial medulla was increased in SHR compared with normotensive rats. Conversely, expression of the novel, non-AT_1_, non-AT_2_, Ang II and III binding site, which was recently discovered, might be decreased in the RVLM and dorsomedial medulla of SHR [45]. They proposed that increased AT_1_ receptor binding in the RVLM might contribute to the hypertension of SHR, whereas reduced radioligand binding to the novel, non-AT_1_, non-AT_2_, angiotensin binding site in the RVLM of SHR might indicate a role for this binding site to reduce blood pressure via its interactions with Ang II and III. 

In this context, the facilitative effect of Ang II on NE release might be an important factor in the excitation of sympathetic tone in the central nervous system, although further studies should be performed to assess more thoroughly the precise roles of the different types of Ang II receptors in the regulation of central sympathetic nerve activity in hypertension. 

### 3.2. Angiotensin-Converting Enzyme Inhibitors

All components of the RAS have been identified in the brain, and the brain RAS might actively participate not only in blood pressure elevation, but also in target organ damages [7–9, 46]. It is proposed that the inhibition of brain ACE activity may be associated with blood pressure reduction induced by ACE inhibitors (ACEIs). Captopril, a widely accepted ACEI, may block the conversion of Ang I to Ang II and has been used as an effective antihypertensive agent in both human and experimental hypertension [47, 48]. Several studies have provided evidence that the distribution of the target sites for ACEIs might be widespread [49, 50]. It was demonstrated that captopril administered centrally significantly lowered blood pressure in intact conscious SHR [51]. Intracerebroventricular administration of captopril significantly suppressed the pressor responses to Ang I given by the same route in SHR [52]. Baum et al. [53] also observed the attenuation of pressor responses to intracerebroventricular Ang I by ACEI in conscious rats. It was shown that oral administration of captopril caused the inhibition of brain ACE activities [50, 54, 55]. It was demonstrated that the ACE activities in various tissues 1 hour after oral administration of several ACEIs and that captopril significantly reduced the ACE activities in aorta, heart, kidney, lung, and brain. Berecek et al. [56] showed that chronic intracerebroventricular injection of captopril attenuated the development of hypertension in young SHR in association with a depression in whole animal reactivity to vasoactive agents and an increased baroreflex sensitivity. It was also observed that central administration of captopril produced a significant depression in vascular reactivity to vasoconstrictor agents in the isolated perfused kidney of SHR in vivo [57]. The findings propose the hypothesis that central action of captopril might be, at least in part, related to vascular relaxation. 

The different structures of ACEIs may influence their tissue distribution and routes of elimination. Cushman et al. [55] examined the effects of various ACEIs on brain ACE activity in SHR. They showed that not only captopril, but also zofenopril produced a modest, short-lasting inhibition of ACE activity in the SHR brain. On the other hand, fosinopril, lisinopril, and SQ 29,852 had delayed but long-lasting inhibitory actions, and ramipril and enalapril showed no effects. More studies are necessary to determine the distribution, binding activity to ACE, and metabolism of each ACEI in the brain.

In an in vitro study presented previously, we showed that captopril significantly inhibited the stimulation-evoked NE release in slices of rat hypothalamus and medulla oblongata [58], as well as in peripheral tissues, such as rat mesenteric arteries [59]. It might be possible that the inhibition of NE release by captopril might be partially due to a reduction in Ang II formation in the central nervous system. The modulation of central NE release by captopril might reduce the sympathetic outflow to the periphery, which could partially explain the hypotensive effects of the ACEI. Recently, Bolterman et al. [60] showed that captopril selectively lowered Ang II, oxidative stress, and endothelin in SHR. On the other hand, it was demonstrated that captopril had an asymmetrical effect on the angiotensinase activity in frontal cortex and plasma of SHR [61]. It might be possible that multiple neuroendocrine actions of ACEIs in the brain could, at least in part, contribute to their blood pressure-lowering efficacy in both human and experimental hypertension. 

### 3.3. Angiotensin Receptor Blockers

It was shown that Ang II administered intracerebroventricularly at a dose that induces drinking behavior in rats significantly increased potassium-stimulated release of NE in the hypothalamus [62]. It can be suggested that Ang II is important primarily in pathological states and that NE plays a substantial role in the brain Ang II-induced drinking response. Furthermore, losartan, an angiotensin receptor blocker (ARB), significantly inhibited the potassium-stimulated NE release in the hypothalamus, acting via the AT1 receptor subtype [62]. Averill et al. [63] showed that losartan attenuated the pressor and sympathetic overactivity induced by Ang II and L-glutamate (an excitatory amino acid) in RVLM of SHR. Huang et al. [64] showed that central infusion of an AT_1_ receptor blocker prevented sympathetic hyperactivity and hypertension in Dahl salt-sensitive hypertensive rats on high salt diet. 

Previous studies have reported only a limited ability of systemic ARB to cross the blood brain barrier [65–69]. Gohlke et al. [65] reported that orally applied AT_1_ receptor blocker candesartan suppressed the central responses of Ang II in a dose- and time-dependent manner in conscious rats, indicating that the AT_1_ receptor blocker might effectively inhibit the centrally mediated action of Ang II upon peripheral application. It was also shown that peripherally administered candesartan markedly decreased AT_1_ binding areas outside (subfornical organ and area postrema) and inside (paraventricular nucleus of the hypothalamus and nucleus of the solitary tract) the blood-brain barrier [66, 67]. Unger [68] proposed that candesartan might be the most effective ARB in crossing the blood-brain barrier. On the other hand, Pelisch et al. [69] demonstrated that CV-11974, the active form of candesartan, was undetectable in brain tissue after oral candesartan treatment, suggesting that candesartan may not cross the intact blood-brain barrier in its active form, or may reach the brain at undetectable levels. They proposed the possibility that undetectable levels of ARB in the brain tissue would be enough to modulate the brain RAS. Further studies are required to determine the relationship between the molecular characteristics of ARBs and their properties to cross the blood-brain barrier [69]. 

 In human hypertensive subjects, Esler [70] proposed that the ability of ARBs to antagonize neural presynaptic angiotensin AT_1_ receptors appears to differ markedly between the individual agents in this drug class. Recently, Krum et al. [71] examined whether ARBs inhibited central sympathetic outflow in human subjects. Using the whole body NE spillover method in humans with essential hypertension, they demonstrated that eprosartan and losartan did not materially inhibit central sympathetic outflow or act presynaptically to reduce NE release at existing rates of nerve firing. They concluded that sympathetic nervous inhibition might not be a major component of the blood pressure-lowering action of ARBs in subjects with essential hypertension.

On the other hand, de Champlain et al. [72] showed that the hypotensive action of valsartan may be mediated in part by an inhibition of the sympathetic baroreflex in patients with essential hypertension. It was also demonstrated that valsartan not only decreased blood pressure, but also shifted the baroreflex set point in hypertensive subjects [73]. Additional studies are necessary to determine the potential effects of ARBs on sympathetic nerve activity in the central nervous system of both human and experimental hypertension. 

### 3.4. Direct Renin Inhibitors

Recent years have seen the development of the nonpeptide, orally long-term effective direct renin inhibitor (DRI), aliskiren, which may block the initial stages of the RAS and exert a sustained antihypertensive action in hypertensive subjects [74–76]. 

With regard to the influences of aliskiren on central sympathetic nervous system, Huang et al. [64] examined whether central infusion of aliskiren prevented sympathetic hyperactivity and hypertension in Dahl salt-sensitive hypertensive rats on high salt diet. Intracerebroventricular infusion of aliskiren markedly inhibited the increase in Ang II levels in the cerebrospinal fluid and in blood pressure caused by intracerebroventricular infusion of rat renin. In Dahl-salt sensitive rats, high salt intake increased resting blood pressure, enhanced pressor and sympathoexcitatory responses to air stress, and desensitized arterial baroreflex function. All of these effects were significantly prevented by intracerebroventricular infusion of aliskiren. These results indicated that intracerebroventricular infusions of aliskiren were effective in preventing salt-induced sympathetic hyperactivity and hypertension in Dahl-salt sensitive rats. Because the ARB also exerted similar effects [64], the result strongly confirms the idea that renin in the brain plays an important role in the salt-induced hypertension.

 In a clinical study, it was shown that aliskiren significantly reduced sympathetic hyperactivity and blood pressure in patients with chronic kidney disease (CKD) [77]. However, Fogari et al. [78] reported that the increase in plasma NE evoked by Ca channel blocker, amlodipine, was not reduced by aliskiren addition in hypertensive subjects. It is strongly suggested that DRI might directly inhibit sympathetic neurotransmitter release, and that the hypotensive action of DRI might largely depend on its inhibitory effect on the sympathetic nerve activity in the central nervous system.

### 3.5. (Pro)renin Receptor

Recent evidence indicates that the (pro)renin receptor (PRR), which is a newly discovered member of the brain RAS, might contribute to the pathogenesis of hypertension [79–82]. Li et al. [83] demonstrated that PRR protein was highly expressed in neurons and upregulated in the subfornical organ and the periventricular nucleus in Ang II-dependent hypertensive mice. In addition, they found that PRR knockdown in the brain significantly decreased blood pressure in renin-angiotensinogen transgenic hypertensive mice, which was associated with a decrease in sympathetic tone and improvement of spontaneous baroreflex sensitivity. Furthermore, PRR knockdown was associated with downregulation of the AT_1_ receptors. It is proposed that PRR blockade in the central nervous system might represent a novel approach for the treatment of neurogenic hypertension. 

### 3.6. Mineralcorticoid Receptor

It has been shown that aldosterone as well as Ang II might act within the central nervous system and cause the sympathetic hyperactivity to increase blood pressure [84–87]. Huang et al. [84] proposed that brain aldosterone-mineralcorticoid receptor (MR)-ouabain pathway might have a pivotal role in Ang II-induced neuronal activation and pressor responses. The sympathoexcitatory effect of aldosterone was blocked by intracerebroventricular of MR antagonist [86]. It was also shown that central infusion of aldosteone synthase inhibitor prevented sympathetic hyperactivity and hypertension by central sodium loading [88]. In a clinical study, Raheja et al. [89] showed that MR blockade by spironolactone prevented chlorthalidone-induced sympathetic activation in hypertensive subjects. Blockade of MR receptors and inhibition of aldosterone synthesis in the central nervous system might lead to a reduction in systemic blood pressure, although it remains unclear whether MR receptor blockers might exert greater antihypertensive effects than other RAS inhibitors. 

### 3.7. Oxidative Stress

The involvement of oxidative stress is implicated in the pathogenesis of hypertension. It was demonstrated that superoxide anions in the RVLM, which might generate hydroxyl radicals, were increased in SHR-SP (stroke prone), suggesting that increased oxidative stress would lead to enhancing the central sympathetic outflow [90, 91]. Recent evidence indicates the possible link between Ang II and NADPH oxidase-derived oxidative stress in the central nervous system [92, 93]. Mertens et al. [93] proposed that AT_1_ receptors might activate the NADPH oxidase complex which could be the most important source of reactive oxygen species (ROS) and suggested that the produced superoxide anion would be converted into H_2_O_2_ by superoxide dismutase or combine with NO to generate peroxynitrite, thereby decreasing NO-bioavailability and promoting lipid and protein oxidation. Additional studies are necessary in order to further unravel the implications of oxidative stress in the Ang II-induced neurotoxicity in the brain. 

## 4. Renin Angiotensin System and Other Neurotransmitter Release in the Central Nervous System

### 4.1. Dopamine Release

There is increasing evidence to suggest that dopaminergic nerve activity in the central nervous system may play a crucial role in the regulation of blood pressure [94–102]. Recent evidence has suggested a functional interaction between brain angiotensin mechanisms and dopamine neurons, although the influences of the RAS on central dopaminergic activities in hypertension are controversial [94–102]. 

It has been demonstrated that ACE is localized throughout the brain and, importantly, is found in neurons of striatum and nigrostriatal tract [48]. In addition, there has been increasing evidence in favor of the involvement of nigrostriatal dopaminergic systems in the pathogenesis of hypertension. Linthorst et al. [95] reported that substantia nigra lesions caused a profound attenuation of the development of hypertension in SHR. Brown et al. [103] showed that the Ang II-induced dopamine release was completely blocked by the AT_1_ receptor antagonist, losartan, but not by the AT_2_ receptor antagonist, PD 123177, suggesting that Ang II acting via the AT_1_ receptor subtype might facilitate the release of dopamine, in the rat striatum. By using the microdialysis method, Mendelsohn et al. [104] showed that administration of Ang II into the rat striatum caused an increase in the release of dihydroxyphenylacetic acid (DOPAC), a dopamine metabolite, and proposed that dopamine release was under a tonic facilitative influence of Ang II.

On the other hand, it has been proposed that the nigrostriatal dopaminergic system may mediate baroreflex sensitivity in rats because both striatal dopamine release and baroreflex responses produced by phenylephrine and carotid occlusion were attenuated by lesions of the nigrostriatal dopaminergic pathway [105]. It has also been reported that centrally administered Ang II increased exploratory behavior in rats and that this effect was antagonized by the dopamine receptor antagonist sulpiride. The finding indicated that Ang II potentiated central dopaminergic effects and dopamine-mediated behavioral responses [106]. Banks et al. [107] demonstrated that the ACE inhibitor captopril inhibited apomorphine-induced oral stereotypy in rats. The observation suggests that a decrease in Ang II formation caused by the ACE inhibitor could block nigrostriatal output. In an in vitro study, we examined the effects of captopril on the release of dopamine, and further determined a possible role of RAS in the regulation of dopaminergic neurotransmission in the central nervous system [108]. We showed that captopril inhibited the release of dopamine in the rat striatum in a dose-dependent manner [108]. Although the mechanisms underlying the neurosuppressive effects of captopril remain to be elucidated, the finding suggests that the inhibition of dopaminergic neurotransmission may be related to the central action of the ACEI.

Jenkins et al. [109] observed that AT_1_ receptors have been identified on dopamine containing cells in the substantia nigra and striatum of human brain using receptor autoradiography. Mertens et al. [110] showed that AT_1_ and AT_2_ receptors might exert an opposite effect on the modulation of DA synthesis in the striatum. Speth and Karamyan [111] demonstrated that the novel, non-AT_1_, non-AT_2_ binding site for angiotensin peptides as a mediator of nontraditional actions of Ang II, could have a role in the stimulation of dopamine release from the striatum.

 Several studies have been made to elucidate the possible link between the brain RAS and the dopaminergic cell death in Parkinson's disease [93, 112, 113]. One hypothesis is that AT_1_ receptors might play a pivotal role in Parkinson's disease. As mentioned above, the stimulation of AT_1_ receptors might lead to the activation of the NADPH oxidase complex and the generation of ROS [92, 93]. Mertens et al. [93] proposed that AT_2_ receptor agonists alone or in combination with AT_1_ receptor blockers might effectively improve the pathological conditions in Parkinson's disease. It was also shown that chronic treatment of ACEI increased striatal dopamine content in the MPTP-mouse [112]. On the other hand, it was demonstrated that administration of PRR blocker significantly decreased 6-hydroxydopamine-induced dopamine cell death in the cultures, suggesting that the potential neuroprotective strategies for dopamine neurons in Parkinson's disease should address not only Ang II but also PRR signaling [113]. 

In this context, it can be speculated that the striatal dopaminergic system may actively participate in the control of blood pressure and behavioral responses, although further studies should be performed to assess the precise role of the RAS in the regulation of central dopaminergic nerve activity and its modulation by the RAS inhibitors.

### 4.2. Acetylcholine Release

Previous studies have shown that the central cholinergic system may also actively participate in blood pressure control [114–123]. Buccafusco and Spector [114] demonstrated that cholinergic stimulation of the central nervous system by direct receptor agonists produced pressor responses involving activation of central muscarinic receptor sites. It was shown that pressor responses induced by intracerebroventricular injection of Ang II were significantly blocked by hemicholinium-3 (an inhibitor of Ach synthesis), which may indicate a possible interaction between the cholinergic nervous system and the RAS in the brain [115]. Vargas and Brezenoff [116] also reported that the decrease in Ach content of the hypothalamus, striatum, and brainstem caused by hemicholinium-3 was associated with a reduction in systemic blood pressure in SHR. In an in vitro study, we have shown that captopril significantly inhibited the stimulation-evoked Ach release in rat striatum [108]. The finding may propose the hypothesis that the inhibition of central cholinergic activity could contribute, at least in part, to the hypotensive mechanisms of captopril.

 Recently, the possible link between the brain RAS and the pathophysiology of Alzheimer's disease has been documented. Tota et al. [124, 125] observed that perindopril and candesartan significantly ameliorated the scopolamine-induced impairment in memory, cerebral blood flow, and cholinergic function in mice. In addition, they suggested that activation of AT_1_ receptors might be involved in the scopolamine-induced amnesia and that AT_2_ receptors could contribute to the beneficial effects of the RAS inhibitors. The findings might propose the idea that inhibition of the brain RAS in hypertensive subjects would be neuroprotective against Alzheimer's disease, although further studies are necessary to assess more thoroughly the relationships between the RAS and central cholinergic nerve activity and their roles in the regulation of blood pressure and neurological functions. 

### 4.3. Vasopressin Release

Brain RAS also modulates the cardiovascular and fluid-electrolyte homeostasis by interacting hypothalamic-pituitary axis and vasopressin release [126, 127]. It was shown that the angiotensinogen-deficient rats had lower plasma levels of vasopressin and an altered central vasopressinergic system [128, 129]. It was also demonstrated that PRR receptor knockdown significantly reduced AT_1_ receptors and vasopressin levels in the renin-angiotensinogen double-transgenic hypertensive mice [83], suggesting that the brain RAS might have a pivotal role in the regulatory mechanisms of vasopressin neurotransmission.

### 4.4. Glutamate/GABA (*γ*-Aminobutyric Acid) Release

The role of the brain RAS on glutamate/GABA neurotransmission has also been described [130–132]. It was shown that bilateral microdialysis of the AT_1_ receptor blocker, ZD7155, into the RVLM significantly decreased glutamate and increased GABA levels [130]. In contrast, administration of AT_2_ receptor blocker, PD 123319, increased glutamate and decreased GABA levels within the RVLM [131]. Fujita et al. [132] demonstrated that administration of candesartan suppressed ischemia-induced increases in the extracellular glutamate with a concomitant reduction in the production of ROS in the retinal ischemia-reperfusion injury model of the rat, indicating that candesartan might protect neurons by decreasing extracellular glutamate after reperfusion and by attenuating oxidative stress via a modulation of the AT_1_ receptor signaling. These findings suggested that the RAS might have a crucial role in the regulation of cardiovascular and neurological functions by modulating glutamate/GABA release in the brain.

## 5. Conclusion

**Figure 1:**
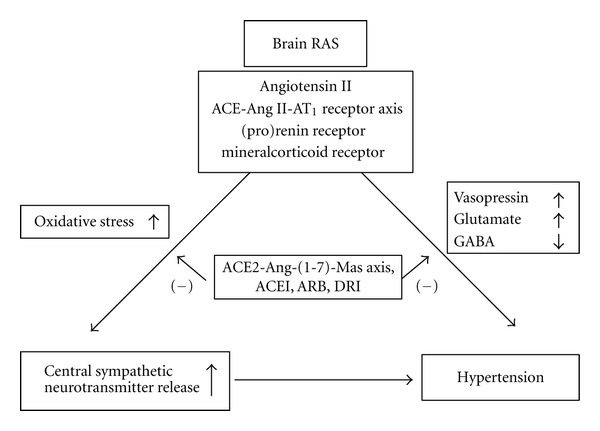
Schematic demonstration of the possible relationship between the brain RAS and sympathetic neurotransmitter release in hypertension. RAS: renin-angiotensin system, ACE: angiotensin converting enzyme, ACEI: angiotensin converting enzyme inhibitor, ARB: angiotensin receptor blocker, DRI: direct renin inhibitor, GABA: *γ*-aminobutyric acid, (–): inhibition.

All components of the RAS have been identified in the central nervous system, and the brain RAS may regulate blood pressure by modulating sympathetic nerve activity. It has been proposed that the RAS may have a stimulatory influence on the sympathetic nervous system. The brain RAS may augment presynaptic facilitation of sympathetic neurotransmitter release and enhance the central sympathetic outflow. In the present paper we discussed the relationship between the brain RAS and sympathetic neurotransmitter release in hypertension (Figure 1). Ang II strongly potentiates sympathetic neurotransmitter release in the central nervous system. In contrast, the inhibitors of the RAS, such as ACEIs, ARBs, and DRIs might suppress sympathetic hyperactivity in the brain. The release of vasopressin, glutamate, and GABA could also be altered by the RAS inhibition. Although the clinical significance of the modulation of central sympathetic neurotransmitter release by the RAS inhibitors is not fully understood, the current findings may be consistent with the idea that the neurosuppressive effect could partially contribute to their hypotensive action in hypertension.

Clearly, more studies are required to further evaluate the precise role of the brain RAS in the control of sympathetic nerve activity, blood pressure, and neurological functions. In addition, better knowledge of the cellular mechanisms in the brain RAS could provide useful information concerning the development of a more specific and more physiological approach to hypertensive research.
